# Materials In Paintings (MIP): An interdisciplinary dataset for perception, art history, and computer vision

**DOI:** 10.1371/journal.pone.0255109

**Published:** 2021-08-26

**Authors:** Mitchell J. P. Van Zuijlen, Hubert Lin, Kavita Bala, Sylvia C. Pont, Maarten W. A. Wijntjes

**Affiliations:** 1 Perceptual Intelligence Lab, Delft University of Technology, Delft, The Netherlands; 2 Computer Science Department, Cornell University, Ithaca, New York, United States of America; University of Jordan, JORDAN

## Abstract

In this paper, we capture and explore the painterly depictions of materials to enable the study of depiction and perception of materials through the artists’ eye. We annotated a dataset of 19k paintings with 200k+ bounding boxes from which polygon segments were automatically extracted. Each bounding box was assigned a coarse material label (e.g., fabric) and half was also assigned a fine-grained label (e.g., velvety, silky). The dataset in its entirety is available for browsing and downloading at materialsinpaintings.tudelft.nl. We demonstrate the cross-disciplinary utility of our dataset by presenting novel findings across human perception, art history and, computer vision. Our experiments include a demonstration of how painters create convincing depictions using a stylized approach. We further provide an analysis of the spatial and probabilistic distributions of materials depicted in paintings, in which we for example show that strong patterns exists for material presence and location. Furthermore, we demonstrate how paintings could be used to build more robust computer vision classifiers by learning a more perceptually relevant feature representation. Additionally, we demonstrate that training classifiers on paintings could be used to uncover hidden perceptual cues by visualizing the features used by the classifiers. We conclude that our dataset of painterly material depictions is a rich source for gaining insights into the depiction and perception of materials across multiple disciplines and hope that the release of this dataset will drive multidisciplinary research.

## Introduction

Throughout art history, painters have invented numerous ways to depict the three-dimensional world onto flat surfaces [1–4]. Unlike photographers, painters are not limited to optical projection [5, 6] and therefore paintings have more freedom. This means that a painter can directly modify and manipulate the 2D image features of the depiction. When doing so, a painter’s primary concern is not whether a depiction is optically or physically correct. Instead, a painting is explicitly designed for human viewing [7, 8]. The artist does not copy a retinal image [9] (which would make the painter effectively a biological camera) but may apply techniques such as iteratively adapting templates until they ‘fit’ perceptual awareness [10].

As a result of this, the depiction contained can deviate from reality [6]. On one hand, this makes paintings unsuited as ecological stimulus [11]. On the other hand, as Gibson acknowledges, paintings are the result of endless visual experimentation, and therefore, indispensable for the study of visual perception.

The depiction and perception of pictorial space in paintings [1–5] has historically received more attention than the depiction and perception of materials. It has previous been found that human observers are able to visually categorize and identify materials accurately and quickly for both photos [12–14] and paintings [15]. Furthermore, for these painted materials, we can perceive distinct material properties such as glossiness, softness, transparency, etc [15–17]. A single material category (e.g., fabric) can already display a large variety of these material properties, which demonstrates the enormous variation in visual appearance of materials. This variation in materials and material properties has received relatively little attention. In fact, the perceptual knowledge that is captured in the innumerable artworks throughout history can be thought of as the largest perceptual experiment in human history and it merits detailed exploration.

### A simple taxonomy of image datasets

To explore material depictions within art there is a need for a dataset that relates artworks to material perception. Therefore, in this study, we create and introduce an accessible collection of material depictions in paintings, which we call the Materials in Painting (MIP) dataset. However, the use and creation of art-perception datasets is of broader interest.

We propose a simple taxonomy of three image dataset usages: 1) perceptual, 2) ecological and, 3) computer vision usage. In the remainder of the introduction below, we will contextualize our dataset within this taxonomy by first discussing existing image and painting datasets as well as the benefits our MIP dataset can provide for each of these three dataset usages. This shall be followed by a detailed description of the creation of the MIP dataset in the method section. Finally, we perform and discuss several small experiments that exemplify the utility of the MIP datasets for each of three dataset usages discussed.

#### Perceptual datasets

To understand the human visual system, stimuli from perceptual datasets can be used in an attempt to relate the evoked perception to the visual input. We can roughly categorize three types of stimuli used for visual perception: natural, synthetic and manipulated.

The first represent ‘normal’ photos of objects, materials and scenes as they can be found in reality. Experimental design with such stimuli often attempts to relate the evoked perceptions to natural image statistics within the images or physical characteristics of the contents captured in the images. Some examples of uses of natural stimuli datasets include, but are not limited to, the memorability of pictures in general [18] or more specifically the memorability of faces [19]. In another example, images of natural, but novel objects were used to understand what underlies the visual classification of objects [20].

The second type, synthetic stimuli, are created artificially, such as digital renderings, drawings and paintings. Synthetic stimuli might represent the real world, but often contain image statistics that deviate from natural image statistics. Paintings have for example often been used to study affect and aesthetics [21–23]. In another example [24], used a set of synthetic stimuli to test for memorability of data visualizations.

Both natural and synthetic images can be manipulated, which leads to the third type of stimuli. Manipulated stimuli are often used to investigate the effect of image manipulations by comparing them to the original (natural or synthetic) image. Here the manipulations function as the independent variables. For example [25] created a database of images that contain scene inconsistencies that can be used to study the compositional rules of our visual environment. In another example, a stimulus set consisting of original and texture (i.e., manipulated) versions of animals found that perceived animal size is mediated by mid-level image statistics [26].

The advantage of using manipulated or synthetic images is that perceptual judgments can be compared to some independent variable, which is typically not available for natural images. Paintings are a special case here. They are a synthetic image of a 3D scene that is rendered using oils, pigments and artistic instruments. However, the painting is also a mostly flat, physical object. Retrieving the veridical data is usually impossible for paintings. In other words, objects or materials depicted in photos can often be measured or interacted with in the real world but this is rarely possible for paintings. However, the advantage of using paintings is that it can often be seen, or (historically) inferred, how the painter created the illusory realism. Even if it cannot be seen with the naked eye, chemical and physical analysis can be performed. In [27] a perceptually convincing depiction of grapes was recreated using a 17th century recipe. In this reconstruction, the depiction was recreated by a professional painter one layer at a time, where each layer represents a separate and perceptually diagnostic image feature that together lead to the perception of grapes. The physical limitations of painterly depictions relative to the physical 3D world, such as for example due to luminance compression in paintings [28–31] may lead to systematically different strategies for material depiction. Despite this [15], has shown that the perceptions of materials and material properties depicted in paintings are similar to those previously reported for photographic materials [14].

Therefore, studying paintings in addition to more traditional stimuli like photos or renderings, can enrich our understanding of human material perception. It should be noted that in this paper we focus on the image structure of the painting instead of the physical object. In other words, we focus on what is depicted within paintings and our data and analysis is limited to pictorial perception. In the remainder of this paper, when we mention *paintings*, we mean *images of paintings*.

Throughout history, painters have studied how to trigger the perceptual system and create convincing depictions of complex properties of the world. This resulted in *perceptual shortcuts*, i.e., stylized depictions of complex properties of the world that trigger a robust perception. The steps and painterly techniques applied by a painter to create a perceptual shortcut can be thought of as a perception-based recipe. Following such a recipe results in a perceptual shortcut, which is a depiction that gives the visual system the required inputs to trigger a perception. Many of the successful depictions are now available in museum collections. As such, the creation of art throughout history can be seen as one massive perceptual experiment. Studying perceptual shortcuts in art, and understanding the cues, i.e., features required to trigger perceptions, can give insights into the visual system. We will demonstrate this idea by analyzing highlights in paintings and photos.

#### Ecological datasets

To understand how the human visual system works it is important to understand what type of visual input is given by the environment. Visual ecology encompasses all the visual input and can be subdivided into natural and cultural ecology. Natural ecology reflects all which is found in the physical world. For example, to understand color-vision and cone cell sensitivities it is relevant to know the typical spectra of the environment. For this purpose, hyperspectral images [32, 33] can be used, in this case to investigate color metamers (perceptually identical colors that originate from different spectra) and illumination variation. In another example, a dataset of calibrated color images were used to understand color constancy [34] (the ability to discount for chromatic changes in illumination when inferring object color). The SYNS database was used to relate image statistics to physical statistics [35]. Another dataset contains photos taken in Botswana [36] in an area that supposedly reflects the environment of the proto-human and was used to investigate the evolution of the human visual system. Spatial statistics of today’s human visual ecology are clearly different from Botswana’s bushes as most people live in urban areas that are shaped by humans. For example, a dataset from [37] was used to compute the distribution of spatial orientations of natural scenes [38].

The content depicted within paintings only loosely reflects the *natural* visual ecology, but they do strongly represent *cultural* visual ecology. They have influenced how people see and depict the world and have influenced visual conventions up to contemporary cinematography and photography. Both perceptual scientists and art historians have looked for and studied compositional rules and conventions within art. A good example is the painterly convention that light tends to originate from the top-left [39, 40], which is likely related to the human light-from-above prior [41–44].

New developments in cultural heritage institutions have made the measurement and study of paintings much more accessible. In recent years the digitization of cultural heritage has led to a surge in publicly available digitized art works. Many individual galleries and art institutions have undertaken the admirable task to digitize their entire collection, and have often make a portion, if not the whole collection digitally available with no or minor copyright restrictions. The availability of digitized art works, combined with advancements in image analysis algorithms, has lead to Digital Art History, which concerns itself with the digitized analysis of artworks by for example analyzing artistic style [45] and beauty [46], or local pattern similarities between artworks [47]. In [48], the authors for example developed a system that automatically detects and extracts garment color in portraits, which can for example be used for the digital analysis of historical trends within clothes and fashion.

Crowley and Zisserman [49] pointed out that art historians often have the unenviable task of finding paintings for study manually. With an extensive dataset of material depictions within art, this task might become slightly easier for art historians that study the artistic depiction of materials, such as for example stone [50, 51]. The ability to easy find fabrics in paintings and it’s fine-grained subclasses such as velvet, silk and lace could be used for the study of fashion and clothes in paintings in general [52, 53] or for paintings from a specific cultural context, such as Italian [54], English and French [55] or even for the clothes worn by specific artists [56]. The human body and it’s skin, which clothing covers, is often studied within paintings [52, 57, 58]. For example, the Metropolitan Museum, published an essay on anatomy in the Renaissance, for which artworks depicting the human nude were used [59]. In this work on anatomy, only items from the Metropolitan Museum were used but with an annotated database of material depictions this could be extended and compared to other museum collections. Furthermore, through material categories such as food and flora category, the MIP could give access to typical artistic scenes such as stillives [60, 61] and floral scenes [62] respectively. It should be noted that ‘stuff’ like skin and food might not appear like a stereotypical material, however in this paper we adhere to the view of Adelson, where each object, or ‘thing’, is considered to consist of some material, i.e., ‘stuff’ [63]. Within this view non-stereotypical ‘stuff’ such as skin and food can certainly be considered as a material.

#### Computer vision datasets

Today, the majority of image datasets originate from research in computer vision. One of the first relatively large datasets representing object categories [64] has been used to both train and evaluate various computational strategies to solve visual object recognition. The ImageNet and CIFAR datasets [65, 66] are regarded to be standard image recognition datasets for the last decade of research on deep learning vision systems.

Traditionally much visual research has been concerned with object classification but recently material perception has received increasing attention [63, 67–69]. A notable dataset that contains material information is OpenSurfaces [67], which contains around 70k crowd-sourced polygon segmentations of materials in photos. The Material In Context database improved on OpenSurfaces by providing 3 million samples across 23 material classes [68]. To our knowledge, no dataset exists that explicitly provides material information within paintings.

The majority of image datasets contain photographs, but various datasets exist that contain artworks. The WikiArt dataset for instance, which is created and maintained by a non-profit organisation, with the admirable goal “to make world’s art accessible to anyone and anywhere” [70]. The WikiArt dataset has been widely used for a variety of scientific purposes [45, 71–74]. The Painting-91 dataset from [75] consists of around 4000 paintings from 91 artists and was introduced for the purpose of categorization on style or artist. More recently, *Art500k* was released, which contains more than 500k low resolution artworks which were used to automatically identify content and style [76] within paintings.

The visual difference introduced by painterly depiction does not pose any significant difficulties to the human visual system, however it can be challenging for computer vision systems as a result of the domain shift [77–79]. Differences between painting images and photographic datasets include for instance composition, textural properties, colors and tone mapping, perspective, and style. As for composition, photos in image datasets are often ‘snapshots’, taken with not too much thought given to composition, and typically intended to quickly capture a scene or event. In contrast, paintings are artistically composed and are prone to historical style trends. Therefore, photos often contain much more composition variation relative to paintings. Within paintings, composition can vary greatly between different styles. The human visual system can *distinguish* styles—for example, Baroque vs. Impressionism—and also implicitly judge whether two paintings are stylistically similar. Research in style or artist classification, as well as neural networks that perform style transfer, attempt to model these stylistic variations in art [45, 80].

Humans can also *discount* stylistic differences, for example, identifying the same person or object depicted by different artists. Similarly, work in domain adaptation [77–79] focuses on understanding objects or ‘stuff’ across different image styles. Models that learn to convert photographs into painting-like or sketch-like images have been studied extensively for their application as a tool for digital artists [80]. Recent work has shown that such neural style transfer algorithms can also produce images that are useful for training robust neural networks [81]. However, photos that have been converted into a painting-like image are not identical to paintings; paintings can contain spatial variations of style and statistics that are not present in photos converted into paintings. Furthermore, painterly convention and composition are not taken into account by style-transfer algorithms.

Depending on the end goal for a computer vision system, it can be important to learn from paintings directly. Of course, when the end goal is to detect pedestrians for a self-driving car, learning from real photos, videos, or renderings of simulations can suffice. However, if the goal is to simulate general visual intelligence, multi-domain training sets are essential. Furthermore, if the goal is to create computer vision systems with a perception that matches human vision, training on paintings could be very beneficial. Paintings are explicitly created by and for human perception and therefor contain all the required cues to trigger robust perceptions. Therefor, networks trained on paintings are implicitly trained on these perceptual cues.

#### The multifaceted nature of datasets

While we have distinguished the broad purposes of datasets and exemplified each with representative datasets, it is important to keep in mind that these datasets can serve multiple goals across the taxonomy. For example, the Flickr Material Database [12] was initially created as a perceptual dataset to study how quickly human participants were capable of recognizing natural materials. However, since then it has also often been used as a computer vision dataset, including by the original authors themselves [82]. In this study paintings are considered as especially interesting as they can be used for perceptual experiments, for digital art history, i.e., cultural visual ecology and can furthermore be used to train and test computer vision networks. The dataset presented in this paper is explicitly designed with this multidisciplinary nature in mind.

## Methods

Here we will first provide a short description of the dataset and the various stages of data collection, followed by an in-depth description of each stage.

Our dataset consists of high-quality images of paintings sourced from international art institutions and galleries. Within these images, human annotators have created bounding box around 15 material categories (e.g., fabrics, stone, etc). We further sub-categorized these material categories into over 50 fine-grained categories (e.g., velvet, etc). Finally, we automatically extract polygon segments for each bounding box. The annotated dataset will be made publicly available. All paintings, bounding boxes, labels, and metadata are available online.

The data collection was executed in multiple stages. Here we give an itemized overview of each stage and subsequently we discuss each stage in depth. The first two stages were conducted as part of a previous study [15], but we provide details here for completeness. Participants were recruited via Amazon Mechanical Turk (AMT). A total of 4451 unique AMT users participated in this study and gave written consent prior to data collection. Data collection was approved by the Human Research (ethics) Committee of the Delft University of Technology and adheres to the Declaration of Helsinki.

- First, we collected a large set of paintings.
- Next, human observers on the AMT platform identified which coarse-grained materials they perceived to be present in each painting (e.g., “is there wood depicted in this painting?”).
- Then, for paintings identified to contain a specific material, AMT users were tasked with creating a bounding box of that material in that painting.
- Lastly, AMT users assigned a fine-grained material label to bounding boxes (e.g., processed wood, natural wood, etc.).

### Collecting paintings

We collected 19,325 high-quality digital reproductions of paintings from 9 online, open-access art galleries. The details of these art galleries are presented in Table 1. Images were downloaded from the online galleries, either using web scraping or through an API. For the majority of these paintings we also gathered the following metadata: title of the work, estimated year of creation and name of the artist.

**Table 1:** List of galleries. A list of all the galleries, the country in which the gallery is located, and the number of paintings downloaded from that gallery.

Gallery Name	Country	Count
The Rijksmuseum	Netherlands	4672
The Metropolitan Museum of Art	USA	3222
Nationalmuseum	Sweden	3077
Cleveland Museum of Art	USA	2217
National Gallery of Art	USA	2132
Museo Nacional del Prado	Spain	2032
The Art Institute of Chicago	USA	936
Mauritshuis	Netherlands	638
J. Paul Getty Museum	USA	399

**Fig 1:**
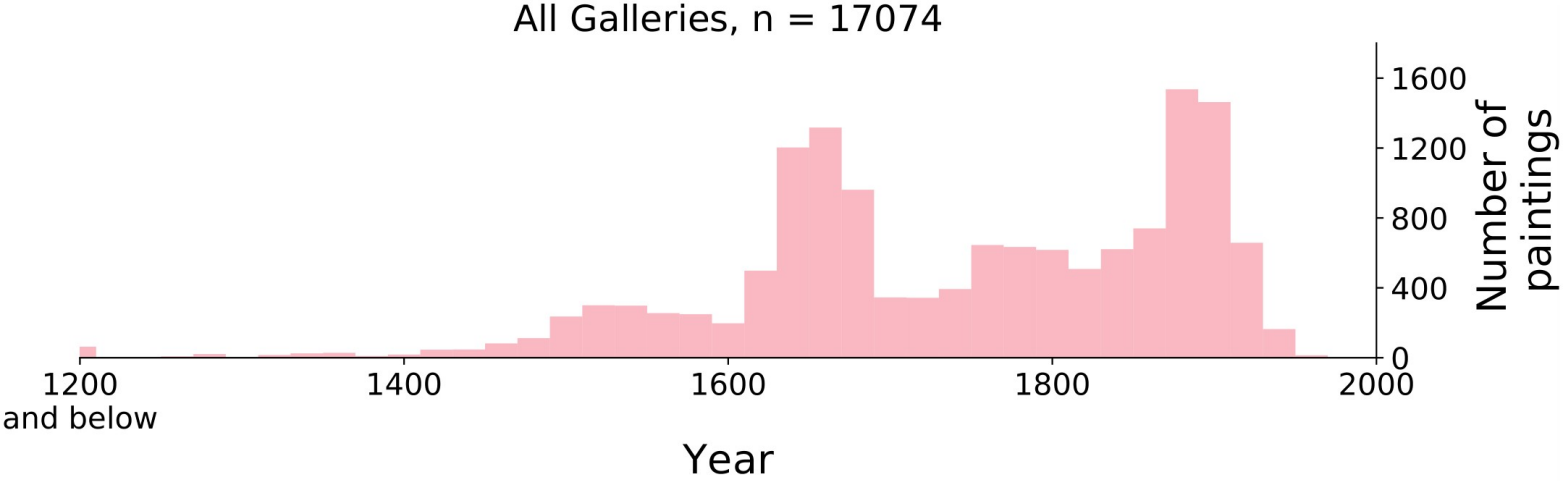
Histogram of the distribution of paintings over time. Each bin equals 20 years.

For 92% of boxes, we also have an estimate of the year of production. These estimates were made by the galleries from which the paintings were downloaded. The distribution of the year of production for all paintings are plotted in Fig 1.

### Image-level coarse-grained material labels

Next, we collected human annotations to identify material categories within paintings. We created a list of 15 material categories: animal, ceramic, fabric, sky, stone, flora, food, gem, wood, skin, glass, ground, liquid, paper, and metal. Our intention was to create a succinct list, that would nevertheless allow the majority of ‘stuff’ within a painting to be annotated. Our list is partially based on material lists used in [12, 14], with which our set has 8 materials categories in common, and partially based on [67], with which our list has 11 material categories in common. Note however minor difference in the category labels; the lists used in [12, 14, 67] has ‘water’, which we have named ‘liquid’ instead.

Our working definition of materials here is heavily influenced by [63, 83], where material does not just refer to prototypical materials that are used as construction materials such as *wood* and *stone*, but also to the ‘stuff’ that makes up ‘things’. For example, few people would consider *banana* as a material, but nevertheless this object has been made up of some type of *banana-material*, which humans are capable of recognizing, distinguishing and estimating physical properties off. In this rational, we have included some less typical ‘stuff’ categories, such as for *food* and *animal*. Note however that we made an exception for *skin*, instead of a more overarching ‘human’ category as one might expect considering based on the previous. We made this choice because of the scientific interest in the artistic depiction [58], perception [84, 85], and rendering of skin directly [86, 87]. Last, we realized that for many paintings a large portion was dedicated to the depiction of the sky or ground ‘stuff’, neither of which are considered a prototypical material, but on average both take up a large portion of paintings. Therefore, in an attempt to more densely annotate the whole region of the painting, we included *sky* and *ground*.

In one AMT task, participants would be presented with 40 paintings at a time and one target material category. In the task, participants were asked if the painting depicted the target material (e.g., *does this painting contain wood?*). They could reply ‘*Yes, the target material is depicted in this painting*.’ by clicking the painting and inversely, by not clicking the painting, participants would reply with ‘*No, the target material is not depicted in this painting*.’. Each painting was presented to at least 5 participants for each of the 15 materials. If at least 80% of the responses per painting claimed that the material was depicted in the painting, we would register that material as present for that painting. In total, we collected 1,614,323 human responses in this stage from 3,233 unique AMT users participating.

### Extreme click bounding boxes

In the previous stage, paintings were registered to depict or not to depict a material. However, that stage does not inform us (1) how often the material is depicted, nor (2) where the material(s) are within the painting.

**Fig 2:**
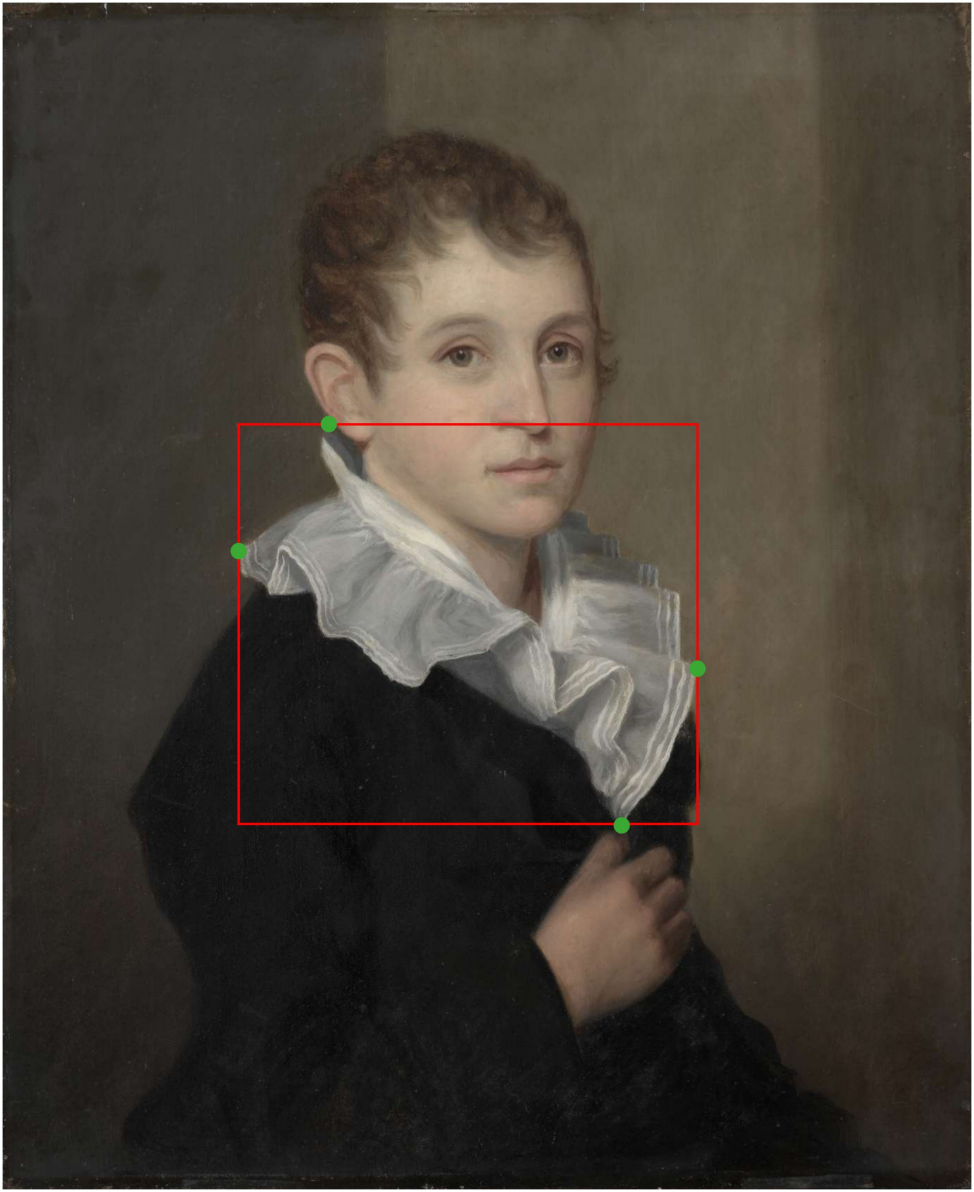
An example of four extreme clicks (marked in green) made by a user on a piece of fabric. These points correspond to the most left, most right, highest and lowest points on the annotated item. The red-line displays the resulting bounding box. *Samuel Barber Clark*, by *James Frothingham*, 1810, Cleveland Museum Of Art, image reproduced under a CC0 license.

We gathered this information on the basis of extreme click bounding boxes. For extreme click bounding boxes, a participant is asked to click on the 4 extreme positions of the material: the highest, lowest, most left-, and most right-wards point [88]. See Fig 2 for an example. In the task, participants were presented with paintings that depicted the target material and tasked to create up to 5 extreme click bounding boxes for the target material.

To make bounding boxes within the task, the participants would use our interface, which allows users to zoom in and out, and pan around the image. The interface furthermore allowed participants to finely adjust the exact location of the extreme points by dragging the points around. Initially, the tasks were open to all AMT workers, but after around 2000 bounding boxes were created by 114 AMT users, with manual inspection, we found that the quality of bounding boxes varied greatly between participants. Therefore, we restricted the work to a smaller number of manually selected participants who were observed to create good bounding boxes. After this restriction, new boxes were manually inspected by the authors, and in a few cases additional participants were restricted due to a deterioration of bounding box quality. Simultaneously additional participants were granted access to our tasks after passing (paid) qualification tasks. As a result, the number of manually selected participants varied between 10 and 20 participants. In total, 227,810 bounding boxes were created by participants.

#### Automatic bounding boxes

While we consider our dataset to be quite larger, it only covers a small but representative portion of art history. It might be required to access materials in paintings that are not part of our dataset. To allow for this, we have trained a FasterRCNN [89] bounding box detector to localize and label material boxes in unlabelled paintings. We use the publicly available implementation from [90] with a ResNet-50 backbone and feature pyramid network (R50-FPN). The model is finetuned from a COCO-pretrained model for 100 epochs using the default COCO hyperparameters from [90]. First we trained the detector on 90% of annotated paintings in the dataset. In section *Automatically detected bounding boxes* in the *Results and demonstrations* section below, we show our evaluation of the network, which was performed on the remaining 10% of annotated paintings. While we created this network to be able to detect paintings outside our dataset, we decided to apply the network on our dataset in order to more densely annotate our paintings. Therefor, after the evaluation, we ran the detection network on the entire set of paintings, i.e., training and testing data, in an attempt to more exhaustively annotate materials within paintings. From the automatic detected bounding boxes we first removed all boxes that scored <50% confidence (as calculated by FasterRCNN). Next, we filtered out automatic boxes that were likely already identified by human annotators, be removing automatic bounding boxes that scored ≥ 50% on intersection over union, i.e., automatic boxes that shared the majority of it’s content with human boxes. This resulted in an additional 94k bounding boxes.

### Fine-grained labels

In this step we supplemented the previously collected material labels with fine-grained material labels (see Table 2). For example, a bounding box labelled as *fabric* could now be labelled as *silk, velvet, fur, etc*. We excluded bounding boxes that were too small (e.g., *width in pixels* × *height in pixels* ≤ 5000) and boxes that were labelled as *sky, ground* or *skin* for which fine-grained categorizations were not annotated. We collected fine-grained labels for the remaining 150,693 bounding boxes. Note that this only concerns the bounding boxes created by human annotations as no automatically detected boxes were assigned a fine-grained material label. For each of these 150,693 bounding boxes, we gathered responses from at least 5 different participants. If the responses reached an agreement of at least 70%, we would assign the agreed upon label to the bounding box. To guide the workers, we provide a textual description for each fine-grained category for them to reference during the task. We did not provide visual exemplars as we did not want to bias the workers into template matching instead of relying on their own perceptual understanding.

**Table 2:** The number of annotated bounding boxes for each coarse- and fine-grained category. Note that not every bounding box is associated with a fine-grained label since participants were not always able to arrive at a consensus. See main text for details.

Coarse-grained	Fine-grained	# Labels
animal		11606
	birds	1822
	reptiles and amphibians	144
	fish and aquatic life	289
	mammals	7752
	insects	155
	other animals	10
ceramic		3641
	brown or red	1088
	white	381
	decorated	289
	other ceramic	14
fabric		31557
	velvety	261
	lace	491
	silky/satiny	1354
	cotton/wool-like	5712
	brocade	96
	fur	27
	other fabric	12
flora		26693
	trees	12851
	vegetables	96
	fruits	1238
	flowers	2515
	plants	3699
food		3690
	cheese	11
	vegetables	107
	fruits	1536
	meat or poultry	183
	bread	127
	seafood	183
	nuts	8
	other	14
gem		10525
	pearls	719
	gemstones	715
	other gems	1
glass		5546
	glass window	2243
	glass container	1003
	other glass	171
ground		2552
liquid		5737
	body of water	4583
	liquid in container	458
	other liquid	172
metal		27708
	colorless metal	2933
	yellowish metal	4435
	brownish or reddish metal	510
	multicolored or other colored metal	215
paper		3167
	paper book	1380
	paper sheets	585
	paper scrolls	114
	other paper	19
skin		32323
sky		12734
stone		23157
	processed stone	9226
	natural stone	9429
wood		26953
	processed wood	12810
	natural wood	10751

We found that it is non-trivial to define fine-grained labels in such a way that they are concise, uniform and versatile (i.e., useful across different scientific domains) while still being recognizable and/or categorizable by naive observers. We applied the following reasoning to select fine-grained labels: first, we tried to divide the materials into an exhaustive list with as few fine-grained labels as possible. For example, for ‘wood’, each bounding box is either ‘processed wood’ or ‘natural wood’. If an exhaustive list would become too long to be useful, we would include an ‘other’ option. For example, for ‘glass’ we hypothesized that the vast majority of bounding boxes would be captured by either ‘glass windows’ or ‘glass containers’. However, to include all possible edge cases such as glass spectacles and glass eyeballs, we included the ‘other’ option.

A possible subset for ‘metal’ we considered was ‘iron’,‘bronze’, ‘copper’, ‘silver’, ‘gold’, ‘other’. However, we feared that naive participants would not be able to consistently categorize these metals. An alternative would be to subcategorize on object-level, e.g. ‘swords’, ‘nails’, etc., but as we are interested in material categorization, we tried to avoid this as much as possible. Thus, for ‘metal’, and for the same reason ‘ceramic’, we required a different method. We chose to subcategorize on color, as often the color for these materials are tied to object identity.

Participants are shown one bounding box at a time and are instructed to choose which of the fine-grained labels they considered most applicable. Additionally, they are able to select a ‘not target material’ option.

We collected over one million responses from 1114 participants. This resulted in a a total of 105,708 boxes assigned with a fine-grained label. See Table 2 for the numbers per category.

## Results and demonstrations

We conducted a diverse set of experiments to demonstrate how our annotated art-perception dataset can drive research across perception, art history, and computer vision. First, we report simple dataset statistics. Next, we organized our findings under the proposed dataset usage taxonomy: perceptual demonstrations, cultural visual ecology demonstrations and computer vision demonstrations.

### Dataset statistics

The final dataset contains painterly depictions of materials, with a total of 19,325 paintings. Participants have created a total of 227,810 bounding boxes and we additionally detected 94k using a FasterRCNN. Each box has a coarse material label and 105,708 also have been assigned a fine-grained material label. The total number of instances per material categories (coarse- and fine-grained) can be found in Table 2. Further analysis of spatial distribution of categories, co-occurences, and other related statistics will be discussed in a following section in the context of visual ecology.

### Perceptual demonstrations

We believe that one of the benefits of our MIP dataset is that selections of the dataset can be useful as stimuli for perceptual experiments. We demonstrate this by performing an annotation experiment to study the painterly depiction of highlights on drinking glasses.

#### Perception-based recipes in painterly depictions

As previously argued, we believe that painterly techniques are a sort of perception-based recipe. Applying these recipes results in a stylized depiction that can trigger a robust perception of the world. Studying the image features in paintings can lead to an understanding of what cues the visual systems needs to trigger a robust perception.

Here we explore a perceptual shortcut for the perceptions of glass by annotating highlights in paintings and comparing these with highlights in photos. In paintings, it has previously been observed that highlights on drinking glasses are typically in the shape of windows, even in outdoor scenes [91]. This highlight-shape can even often be found in contemporary cartoons [92, 93]. This convention can be considered as a perception-based recipe, where the result is a window-shaped highlight that appears to be a robust cue that triggers the perception of gloss for drinking glasses.

We used bounding boxes from our dataset and photographs sourced from COCO [94]. Participants for this study included 3 of the authors, and one lab-member naive to the purpose of this experiment.

*Images*. We used 110 images of drinking glasses, split equally across paintings and photos. First, we selected all bounding boxes in the *glass, liquid container* category in our dataset. From this set, we manually selected drinking glasses, since this category can also contain items such as glass flower vases. Next, we removed all glasses that were mostly occluded, were difficult to parse from the background—for example when multiple glasses were standing behind each other, and removed images smaller then 300x300 pixels. This resulted in a few hundred painted drinking glasses.

Next, we downloaded all images containing cups and wineglasses from the COCO [94] dataset, from which we removed all non-glass cups, occluded glasses, blurry glasses and glasses that only occupy a small portion of the image, and small images. This left us with 55 photos of glass cups and wineglass. Next, we randomly selected 55 segmentations from our painted glass collection. Each image was presented in the task at 650 × 650 pixels, keeping aspect ratio intact.

**Fig 3:**
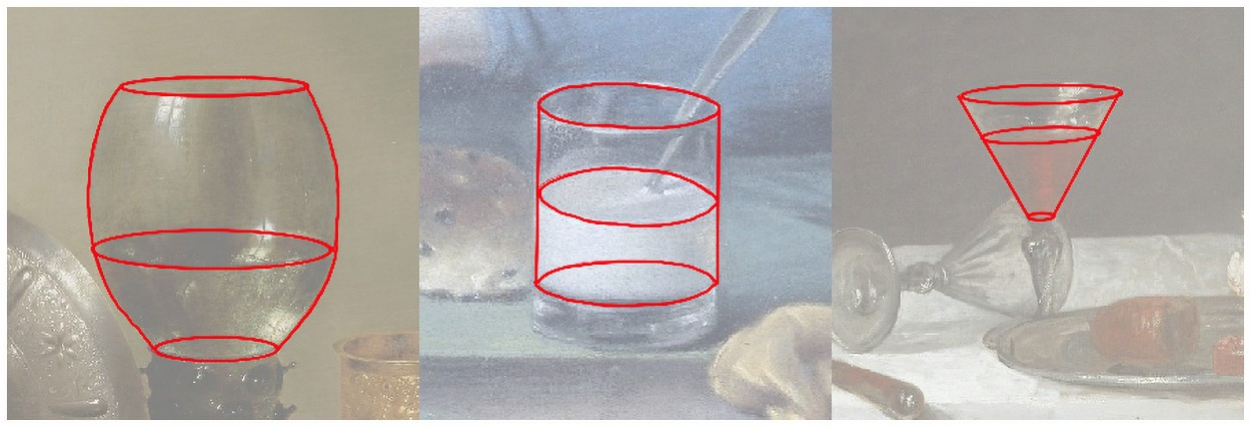
Examples of the three glass shapes. From left to right: spherical, cylindrical and conical. The red geometry annotations were manually created by the authors, and were used to standardize across glasses for the highlight analysis. Paintings used, from left to right: *Portret van een jongen, zittend in een raamnis en gekleed in een blauw jasje*, by *Jean Augustin Daiwaille*. 1840. *Still Life with a gilded Beer Tankard*, by *Willem Claesz. Hed*, 1634. *The White Tablecloth*, by *Jean Baptiste Siméon Chardin*, 1731. The left and middle images courtesy of The Rijksmuseum. The right image courtesy of The Art Institute of Chicago. All images reproduced under a CC0 license.

During this selection phase, we did not base our decision on the shape of the glass. After the experiment, as part of the analysis, we divided the glasses into three shapes, namely spherical, cylindrical, and conical glasses. See Fig 3 for an example of each shape.

*Task*. Participants annotated highlights on drinking glasses using an annotation interface. In the annotation interface, users would be presented with an image on which the annotated geometry was visible. This made it clear which glass should be annotated, in case multiple glasses were visible in the image. Users were instructed to instruct all visible highlights on that glass. Once the user started annotating highlights, the geometry would no longer be visible. Annotations could be made by simply holding down the left-mouse button and drawing on top of the image. Once a highlight was annotated a user could mark it as finished and continue with the next highlight, and eventually move to the next image.

**Fig 4:**
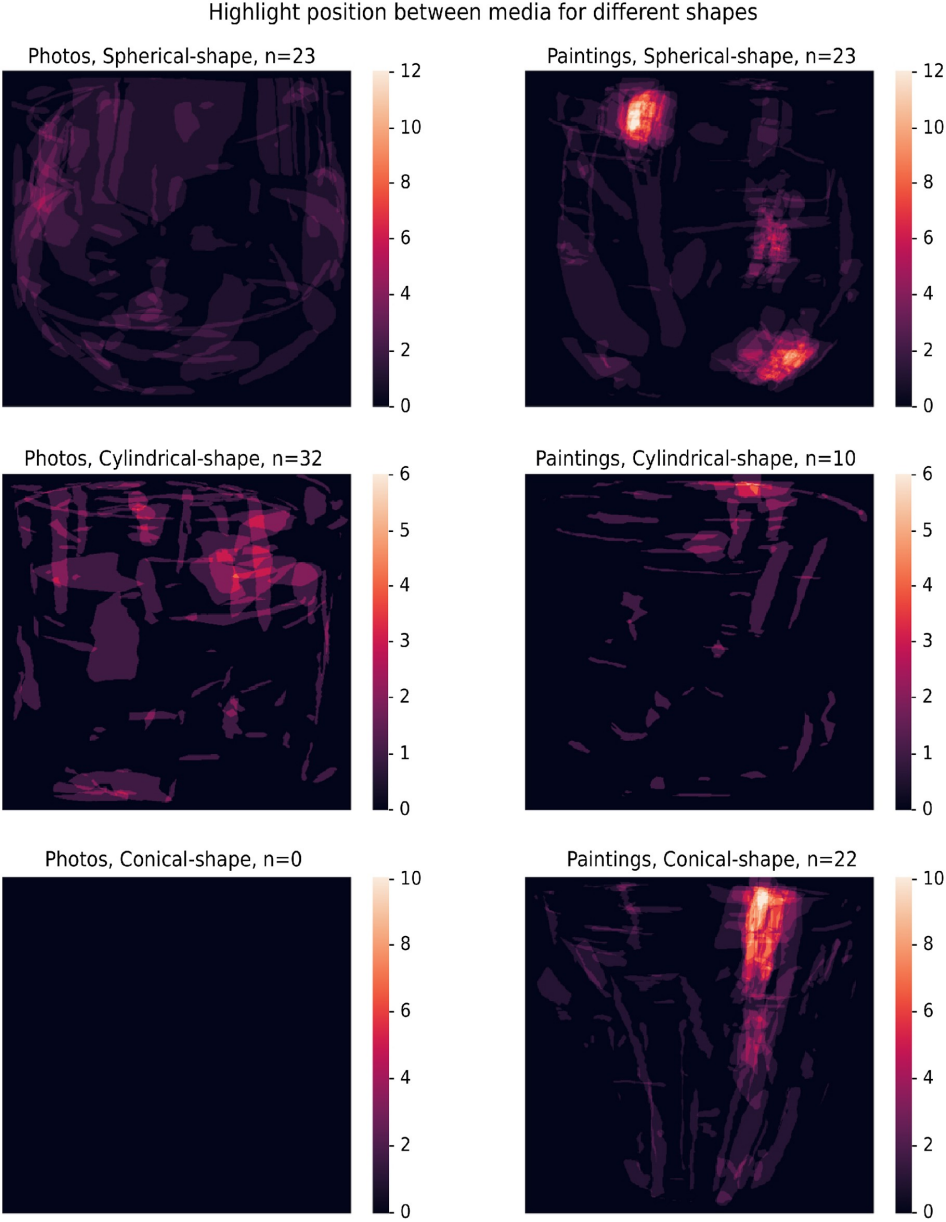
The overlaid highlights created by users, split on media and glass shape. In general, the photographic glass shapes display more variability and do not display a clear pattern. Note that for photos, no stimuli existed with a conical shape in our set which leads to a black image, since there were no highlight-annotations. On the right, for painted glasses, we see clear patterns in the placement of highlights for each glass shape.

*Results*. To compare the highlights between photos and paintings, we resized each glass to have the same maximum width and height, and then overlaid each glass on the center. Initially, we overlaid all images for both types of media (not visualized here) and found the resulting figure quite noisy. However, when we split the glasses on media and shape, clear patterns emerge for painted glasses Fig 4.

As can be seen, painters are more likely to depict highlights on glasses adhering to a stylized pattern, at least for spherical and conical glasses. This pattern of highlights is perceptually convincing and very uniform in comparison with the variation found within reality.

Furthermore, we calculated the agreement between each pair of participants, as the ratio of pixels annotated by both participants (i.e., overlapping area) divided by the number of pixels that was an annotated by either participant (i.e., total area). Averaged across participants, the agreement on paintings (0.33) was around 50% higher relative to the average agreement between participants on photos (0.21). This means that for our stimuli, highlights in paintings are less ambiguous when compared to photos.

### Cultural visual ecology demonstrations

The ecology displayed within paintings are representative of our visual culture. Our dataset consists of paintings spanning 500+ years of art history. This provides a unique opportunity to analyze a specific sub-domain of visual culture, i.e., that of paintings. Here we first analyze the presence of materials in paintings in the *Material presence* section and in the next section we analyse this over time. In *The spatial layout of materials*, we visualize the spatial distributions of materials in our dataset. In the last section, we analyze the automatically detected bounding boxes.

#### Material presence

**Fig 5:**
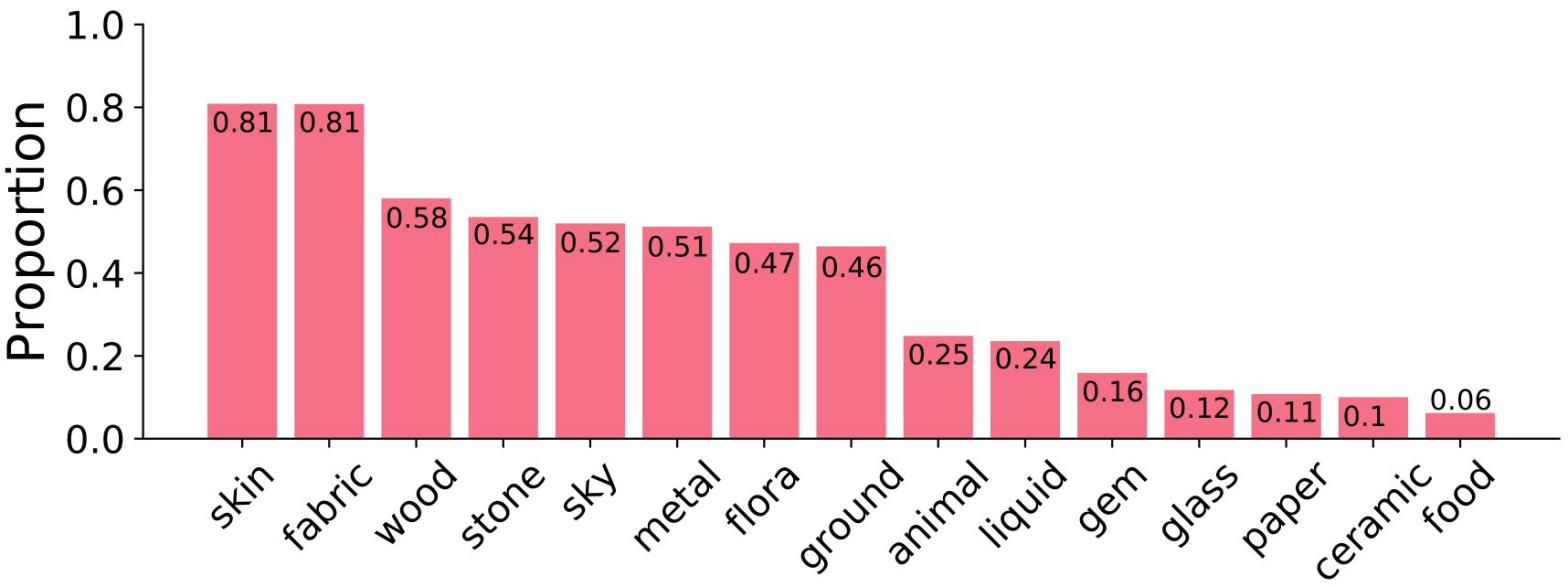
The proportion of paintings in our dataset that depict at least one instance of each material.

Within the 19,325 paintings, participants exhaustively identified the presence of 123,244 instances of 15 coarse materials. In other words, for each painting, participants indicated if each material is or is not present. The distribution of unique materials per painting is normally distributed with an average of 5.7 unique coarse materials present per painting (std = 2.8 materials). The most frequent materials are *skin* and *fabric*. The least frequent are ceramics and food. The relative frequency of each coarse material is presented in Fig 5. We did not exhaustively identify fine-grained materials within paintings, so we will not report those statistics here.

**Fig 6:**
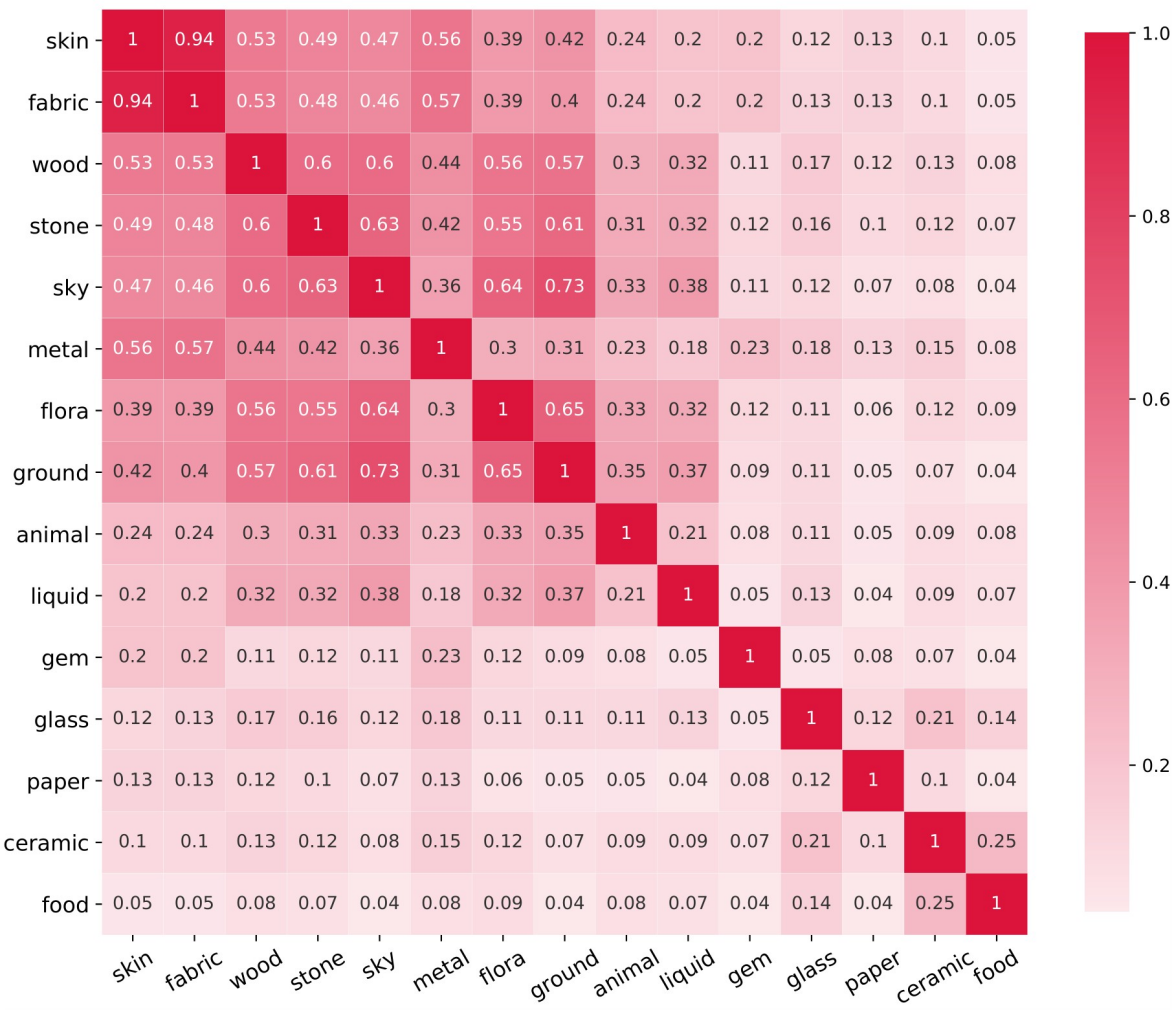
Co-occurrence matrix. Each cell equals the number of paintings where both materials are present divided by the number of paintings where one or the other material is present.

Based on prior knowledge of natural ecology, one might assume that some materials, such as *skin* and *fabric* might often be depicted together in paintings. To quantify the extent to which materials are depicted together, we create a co-occurrence matrix presented in Fig 6, where each cell is the co-occurrence for each pair of materials as the number of paintings where both materials are present, divided by the number of paintings where either (but not both) materials are present. We can see for example, that if *skin* is depicted, there is a 94% change to also find *fabric* in the same painting.

**Fig 7:**
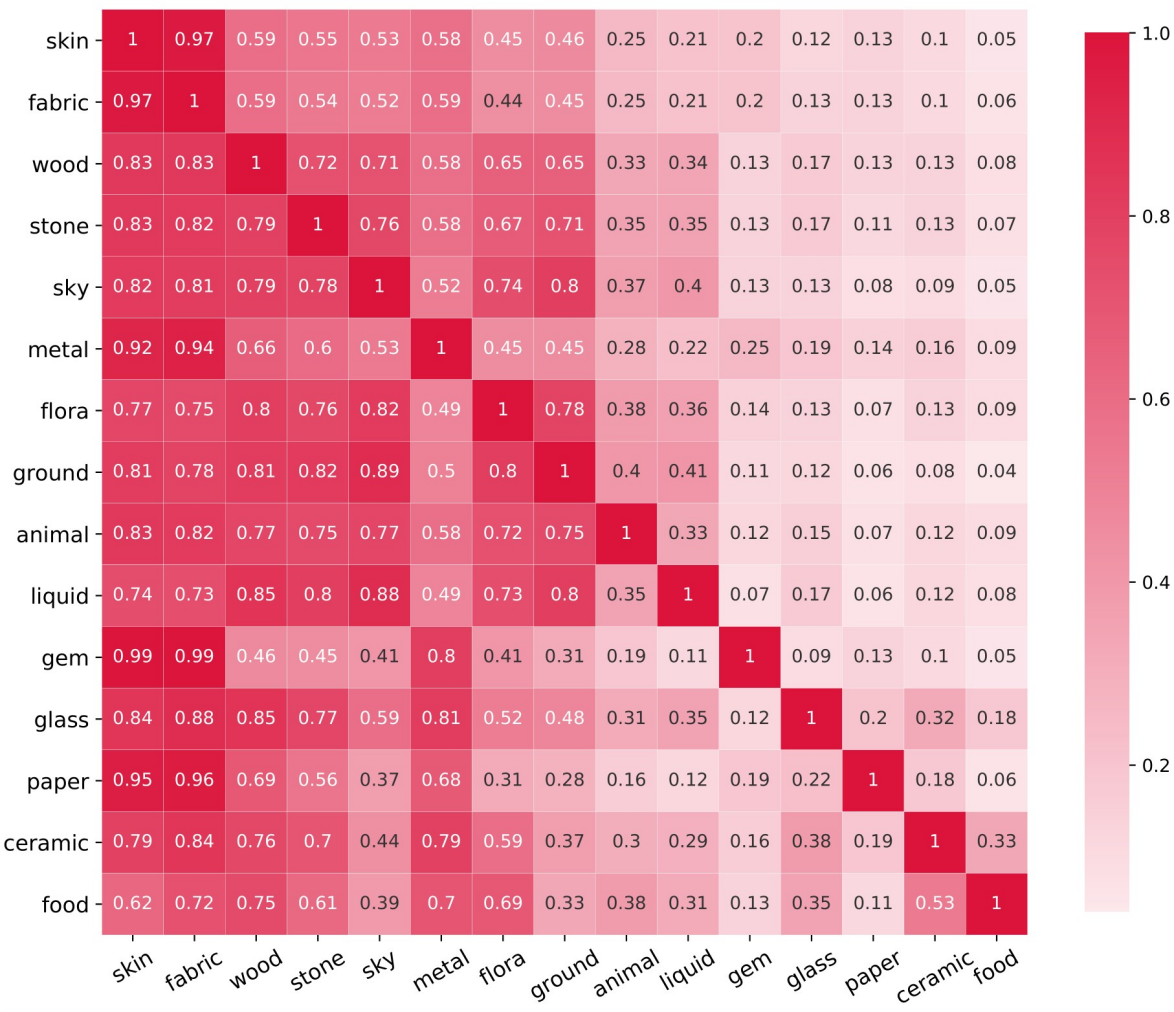
Likelihood matrix. This matrix visualizes the influence a material has on the likelihood of finding another material within the same painting, i.e., if one material on the y-axis is present, then how does this impact the presence of other materials on the x-axis? Calculated as the number of paintings where both materials are present, divided by the number of paintings that contain only one of the materials.

Furthermore, one might expect that the presence of one material can have an influence on another material. For example, one might expect that *gem* might almost always be depicted with *skin*, but that *skin* is only sometimes depicted with *gem*. To quantify these relations, we calculated the occurrence of a material given that another material is present. We visualize this in Fig 7. Here we see that *if gem* is present, *then skin* is found in 99% of the paintings, but that *if skin* is present, *then gem* is found in only 20% of the paintings. The same relationship is true for gem and fabric. This implies that gems are almost always depicted with human figures, however that human figures are not always shown with gems. Another example, when liquid is present, in 85% of the paintings, wood is also present. One might be reminded of typical naval scenes, or landscapes with forests and rivers. Inversely, when wood is present, only 34% of the paintings depict liquid. For *food* and *ceramics*, two materials which are present in less then 10% of paintings, we see that if *food* is present, *ceramics* has a 53% change to be present as well, but the inverse is only 33%. This implies that food is served in, or with, ceramic containers half of the time, but that this is only 1/3rd of what ceramics is used for.

#### Material presence over time

We have previously shown the distributions of materials in paintings in Fig 5. When we created similar distributions (not visualised) for temporal cross-sections, for example for a single century, we found that these distributions were remarkably similar to the average distribution in Fig 5. We used t-tests, to see if the distribution for any century was significantly different from the average distribution in Fig 5 and found no significant effect. This means that despite the changes in stylistic and artistic techniques over time, the distribution of materials (such as in Fig 5 remained remarkably stable over time for the period covered in our dataset.

#### The spatial layout of materials

Paintings are carefully constructed scenes and it follows that a painter would carefully choose the location at which to depict a material. With the knowledge that spatial conventions exists within paintings (e.g., lighting direction [39, 40]), one can assume that these might extend to materials. The average spatial location and extent of materials is visualized by taking the (normalized) location of each bounding box for a specific material and subsequently plotting each box as a semi-transparent rectangle. The result is a material heatmap, where the brightness of any pixel indicates the likelihood to find a material at that pixel. In this section, we limit the material heatmaps to only include the bounding boxes created by human annotators. In the next section, we visualize the material heatmaps for automated boxes too.

**Fig 8:**
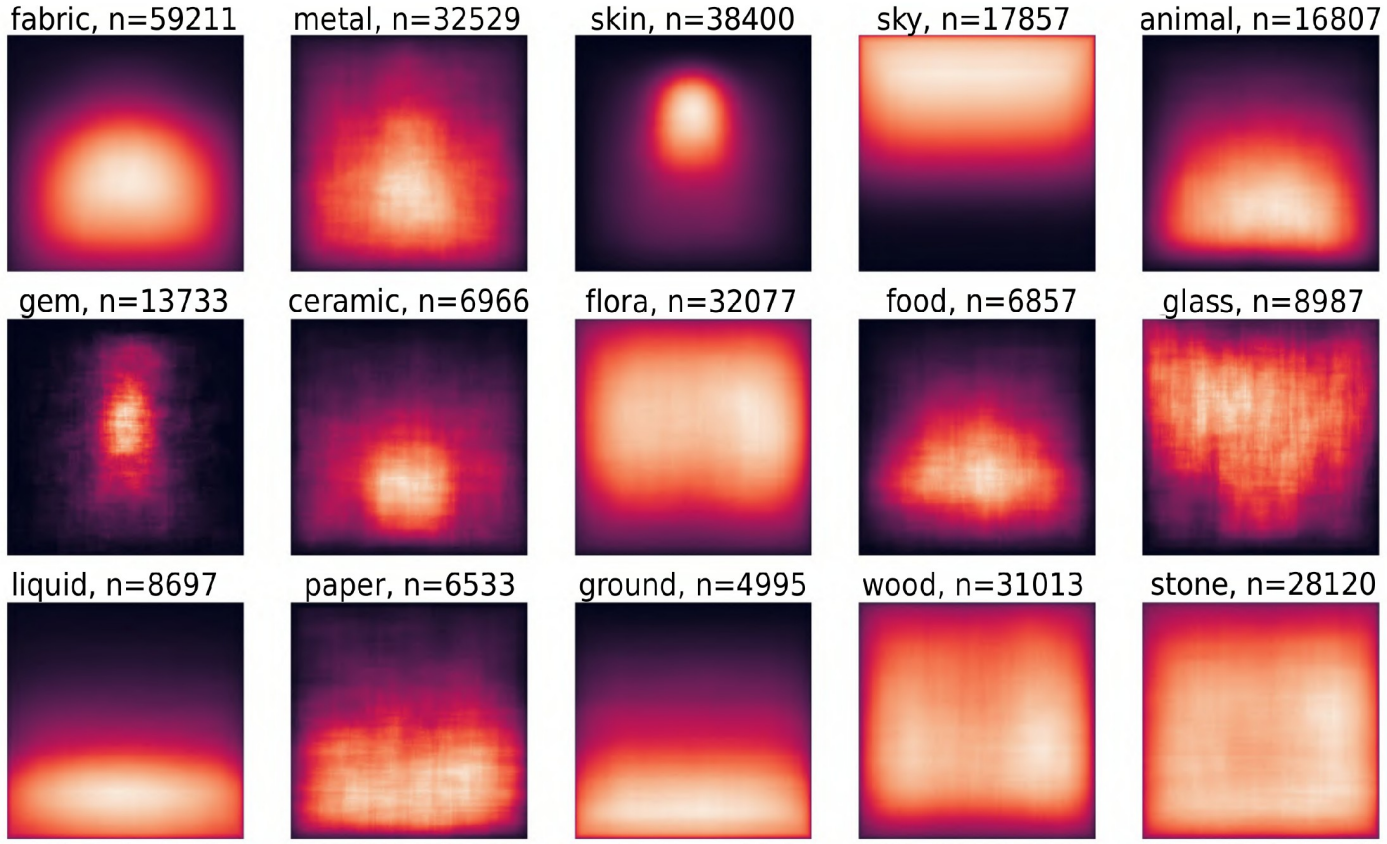
Material heatmaps, which illustrate the likelihood at any given pixel to find the target material at that pixel. Brighter colors indicate higher likelihoods.

**Fig 9:**
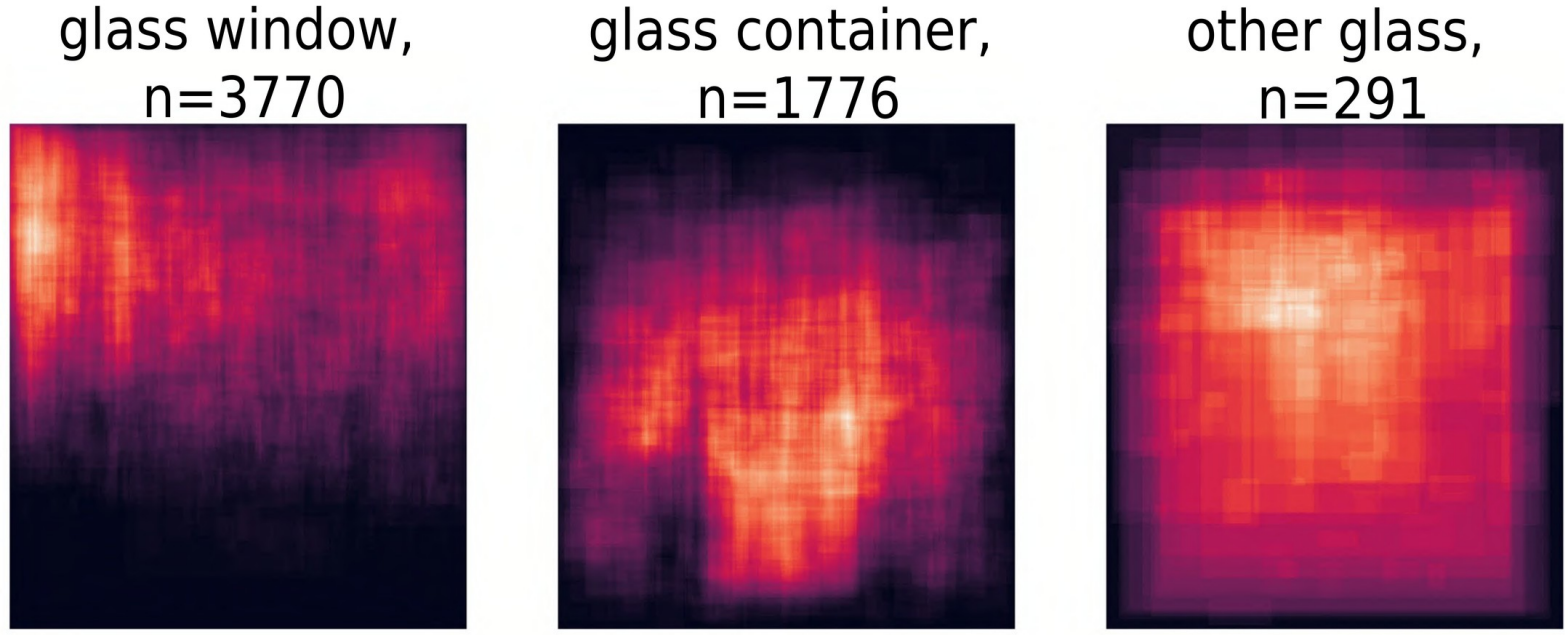
Material heatmaps for glass sub categories. For glass windows, it is interesting to see the clustering in the top-left corner, which is in agreement with the artistic convention of having light come from the top left.

Material heatmaps for the 15 coarse materials are shown in Fig 8. The expected finding that *sky* and *ground* are spatially high and low within images serves as a simple validation or sanity-check of the data. It is interesting to see how *skin* and *gem* are both vertically centered within the canvas. It appears to suggests a face, with necklaces and jewelry adorning the figure. In general, each material heatmap appears to be roughly vertically symmetric. For *glass*, there does however appear to be a minor shift towards the top-left. This might be related to an artistic convention, namely that light in paintings usually comes from a top-left window [39]. When we look at the heatmaps for the sub-categories for glass in Fig 9, we see that it is indeed glass windows that show the strongest top-left bias.

#### Automatically detected bounding boxes

Besides the bounding boxes created by humans, we also trained a FasterRCNN network to automatically detect bounding boxes with 90% of the data as training data. On the remaining unseen 10% of paintings, the network detected 90,169 bounding boxes. We removed those with a confidence score below 50%, which resulted in 24,566 remaining bounding boxes. In the section below, all references to the automated bounding boxes refer to these 24,566 bounding boxes.

**Fig 10:**
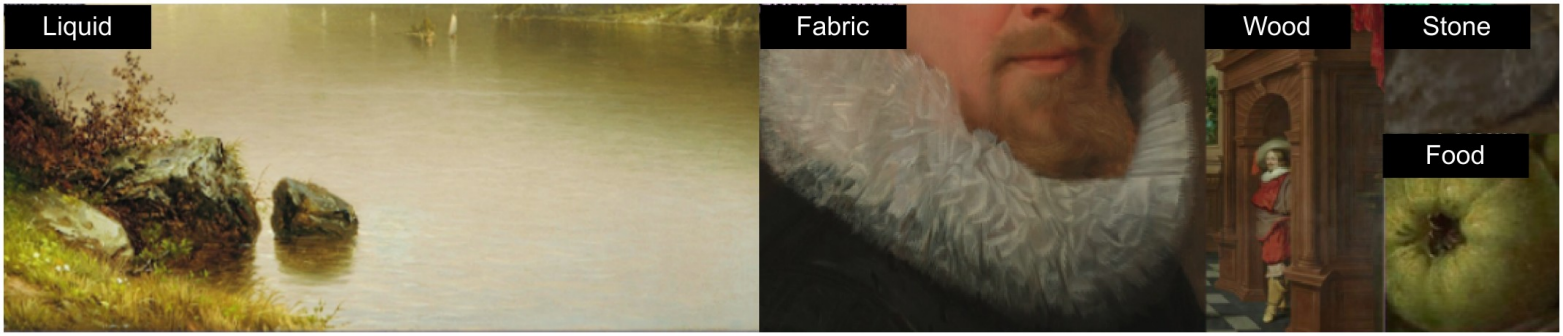
Examples of detected materials in unlabeled paintings. Automatically detecting materials can be useful for content retrieval for digital art history and for filtering online galleries by viewer interests.

**Fig 11:**
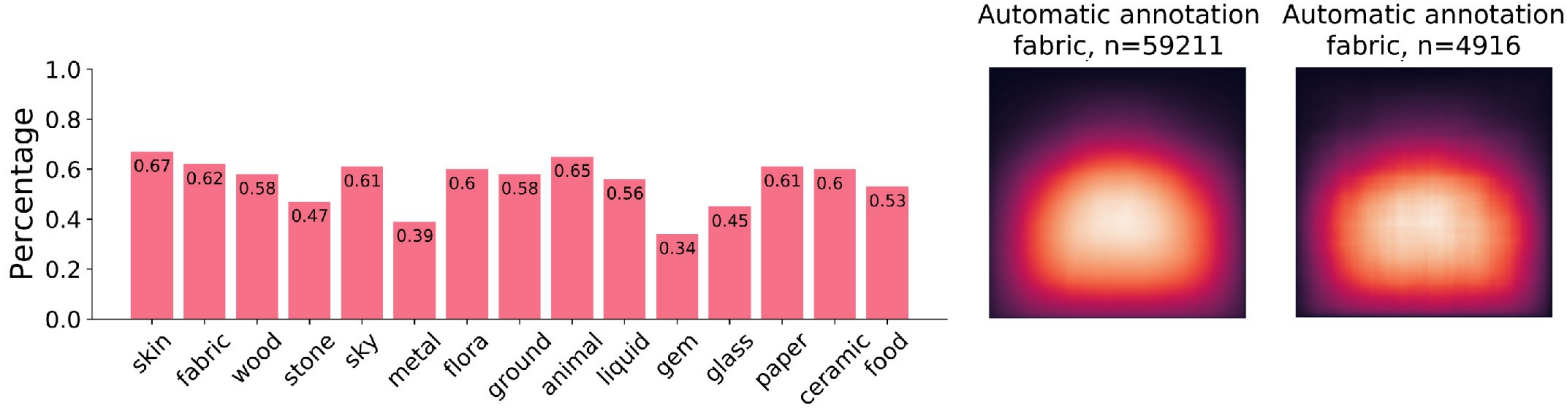
In the bar graph, the accuracy for automatically detected bounding boxes is displayed in the same order as in Fig 5. The values were derived from human quality votes. On the right, we compare the material heatmaps for fabric between the automated and the human annotation bounding boxes. From left to right, top to bottom: *Lake George*, by *John William Casilear*, 1857. *Man with a Celestial Globe*, by *Nicolaes Eliasz Pickenoy*, 1624. *A Seven-Part Decorative Sequence: An Interior*, by *Dirck van Delen*, 1631. *Thomas Howard, 2nd Earl of Arundel*, by *Anthony van Dyck*, 1620. *The Poultry Seller*, by *Cornelis Jacobsz. Delff*, 1631. First and second digital image courtesy of The Metropolitan Museum of Art. Third and last image courtesy of The Rijksmusuem. Fourth image courty of the Getty’s Open Content Program. All images reproduced under a CC0 license.

A qualitative sample of detected bounding boxes is given in Fig 10. Our human bounding boxes are non-spatially exhaustive in nature meaning that not every possible material has been annotated. As a result, the automatically created bounding boxes cannot always be matched against our human annotations and thus we cannot use this to evaluate their quality. In order to validate the automatic bounding box detection, we performed a simple user study to get an estimate of the accuracy per material class, which is visualized in Fig 11. In the user study, a total of 50 AMT participants judged a random sample of 1500 bounding boxes. The 1500 bounding box stimuli were divided into 10 sets of 150 stimuli, where each set contain 10 boxes per course material class. Each individual participant only saw one set and each set was seen by 5 unique participants. Therefore, this can be thought of as 10 experiments, each with 5 participants and 150 stimuli, where participants performed the same task across each set/experiment. The participants were tasked to rate whether each stimuli is either a *good* or a *bad* bounding box, where a *bad* bounding box was defined as either 1) having the wrong material label (e.g., “I see wood, but the label says stone”) or as having a bad boundary where the edges of the bounding box were not near the edges of the material. The order of stimuli was randomized between sets and participants. This leads to a total of 7500 votes, 500 per material classes. The ratio of good to bad votes per material classes can serve as a measure of accuracy, which has been visualized in Fig 11.

The participant agreement averaged across bounding boxes was found to be 80%, i.e., on average 4 out of 5 participants agreed on their rating per bounding box. As a result of the user study, we found a mean average accuracy of 0.55 across participants. While not high, these results are somewhat interesting in that they show that a FasterRCNN model is capable of detecting materials in paintings, without any changes to the network architecture or training hyperparameters. It is certainly promising to see that an algorithm designed for object localization in natural images can be readily applied to material localization in paintings. Likely, the accuracy could be further improved by finetuning the network which we have not done in this paper.

It is interesting to note that the spatial distribution of automatically detected bounding boxes looks very similar to the spatial distribution of the human annotated bounding boxes. We have visualized the material heatmap for one material, fabric, for the automated bounding boxes to show the similarity with the material heatmap for the same material created from human annotation bounding boxes. This has been visualized in the right side of Fig 11

### Computer vision demonstrations

In this section, we will first apply existing segmentation tools designed for natural photographs to extract polygon segmentations. Next, we perform an experiment to demonstrate the utility of paintings for automated material classification.

#### Extracting polygon segmentations

A natural extension of material bounding boxes is material segments [67–69]. Polygon segmentations are useful for reasoning about boundary relationships between different semantic regions of an image, as well as the shape of the regions themselves. However, annotating segmentations is expensive and many modern datasets rely on expensive manual annotation methods [67, 69, 94–96]. Recent work has focused on more cost effective annotation methods (e.g. [97–100]). One broad family of methods to relax the difficulty of annotating polygon segmentations is through the use of interactive segmentation methods that transform sparse user inputs into a full polygon masks.

**Fig 12:**
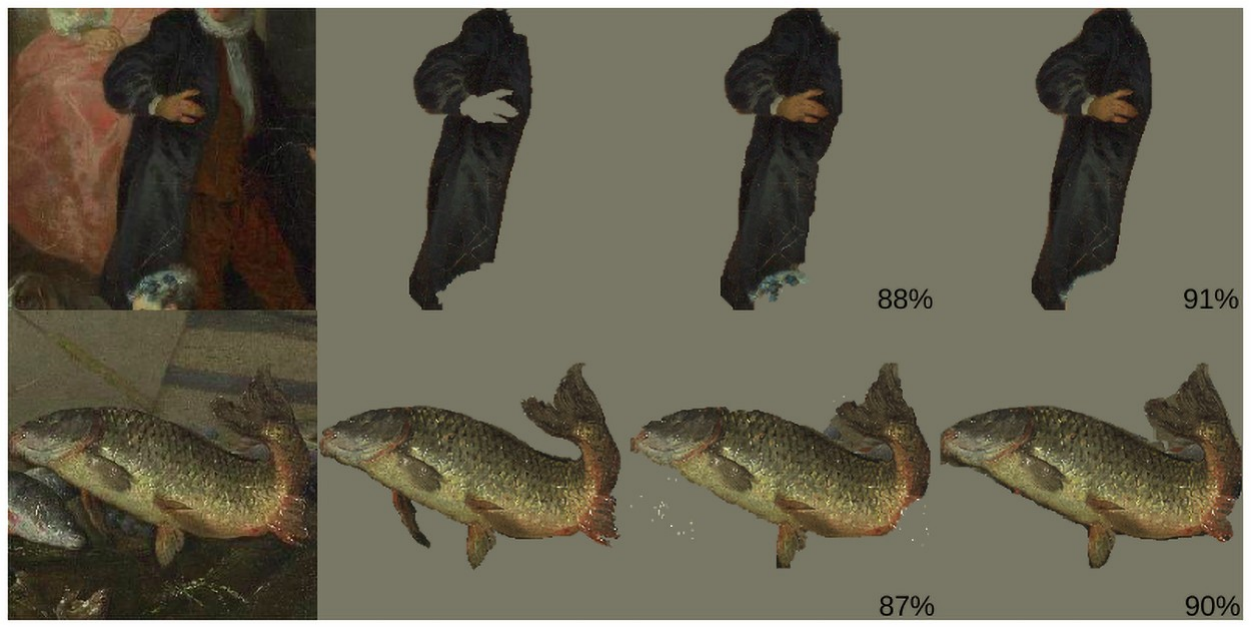
Segmentation visualizations. Left to right: Original Image, Ground Truth Segment, Grabcut Extr Segment, DEXTR COCO Segment. Both Grabcut and DEXTR use extreme points as input. For evaluation, the extreme points are generated synthetically from the ground truth segments. The IOU for each segmentation is shown in the bottom right corner. Top image: *Dance before a Fountain*, by *Nicolas Lancret*, 1724, Digital image courtesy of the Getty’s Open Content Program. Bottom image: *Still life with fish*, by *Pieter van Noort*, 1660, Het Rijksmusuem. Images reproduced under a CC0 license.

For this dataset, we apply interactive segmentation with the crowdsourced extreme clicks as input. To evaluate quality, we compared against 4.5k high-quality human annotated segmentations from [15], which were sourced from the same set of paintings. We find that both image-based approaches like GrabCut (GC) [101] and modern deep learning approaches such as DEXTR [98] perform well. Surprisingly, DEXTR transfers quite well to paintings despite being trained only on natural photographs of objects. The performance is summarized in Table 3. The performance is summarized using the standard intersection over union (IOU) metric. IOU is computed as the intersection between a predicted segment and the ground truth segment divided by the union of both segments. IOU is computed for each class, and mIOU is the mean IOU over all of the classes. Samples are visualized in Fig 12. Segments produced by these methods from our crowdsourced extreme points will be released with the dataset.

**Table 3:** Segmentations from extreme clicks. Grabcut [101] rectangles use bounding-box only initialization as a reference baseline. Grabcut Extr is based on the improved GC initialization from [88] with small modifications: (a) we compute the minimum cost boundary with the cost as the negative log probability of a pixel belonging to an edge; (b) in addition to clamping the morphological skeleton, we also clamp the extreme points centroid as well as the extreme points; (c) we compute the GC directly on the RGB image. DEXTR [98] Pascal-SBD and COCO are pretrained DEXTR ResNet101 models on the respective datasets. Note that Pascal-SBD and COCO are natural image datasets of objects, but DEXTR transfers surprisingly well across both visual domains (paintings vs. photos) and annotation categories (materials vs. objects).

mIOU (%)				
Grabcut Rectangle	Grabcut Extr	DEXTR Pascal-SBD	DEXTR COCO	DEXTR Finetune
44.1	72.4	74.3	76.4	78.4
DEXTR Finetune IOU By Class (%)				
Animal	Ceramic	Fabric	Flora	Food
76.9	86.8	79.1	77.0	87.5
Gem	Glass	Ground	Liquid	Metal
74.4	83.2	69.6	73.0	75.5
Paper	Skin	Sky	Stone	Wood
86.1	78.9	78.5	81.7	67.4

#### Learning robust cues for finegrained fabric classification

The task of distinguishing between images of different semantic content is a standard recognition task for computer vision systems. Recently, increasing attention is being given to “fine-grained” classification, where a model is tasked with distinguishing images of the same coarse-grained category (e.g., distinguishing different species of birds or different types of flora [102–104]). Classifiers for material categories can perform reasonably well on coarse-grained classification by relying on context alone. In comparison, fine-grained classification is more challenging for deep learning systems as contextual clues are often equal within fine-grained classes. For example, one might reason that the material of a shirt might be recognized as fabrics partly because of the context, i.e., being worn by a figure. However, in fine-grained classification context can be held consistent across classes (for example, both velvet shirts and satin shirts are worn). To successfully distinguish between these two fine-grained classes in a context-controlled setting, a classifier should use non-contextual features (at least more so relative to uncontrolled settings).

The rational above leads to two interesting possibilities. First, we hypothesize that the painted depictions of materials can be beneficial for fine-grained classification tasks. Since artistic depictions focus on salient cues for perception, i.e., paintings are explicitly created for and by perception, it is possible that a network trained on paintings is able to learn a more robust feature representation by focusing on these cues.

Second, visualising the features used by a successful fine-grained classifier could potentially lead to the uncovering of latent perceptual cues. For example, in the *Perception-based recipes in painterly depictions* section above, we showed that window-shaped highlights are a robust cue for the perception of gloss on drinking glasses. However, it is assumed that the visual system used many such cues which are as of now unknown. Visualising what cues are used by classifiers might lead to the finding of cues used by the perceptual system.

*Task*. We experimented with the task of classifying cotton/wool versus silk/satin. The latter can be recognized through local cues such as highlights on the cloth; such cues are carefully placed by artists in paintings. To understand whether artistic depictions of fabric allow a neural network to learn better features for classification, we trained a model with either photographs or paintings. High resolution photographs of cotton/wool and silk/satin fabric and clothing (dresses, shirts) were downloaded and manually filtered from publicly available photos licensed under the Creative Commons from Flickr. In total, we downloaded roughly 1K photos. We sampled cotton/wool and silk/satin samples from our dataset to form a corresponding dataset of 1K paintings. We analyzed the robustness of the classifier trained on paintings versus the classifier trained on photos in two experiments below. Taken together, our results provide evidence that a classifier trained on paintings can be more robust than a classifier trained on photographs, and that visualizing these features could lead to discovering perceptual cues utilized by the human visual system.

*Generalizability of classifiers*. Does training with paintings improve the generalizability of classifiers? To test cross-domain generalization, we test the classifier on types of images that it has not seen before. A classifier that has learned robust features will outperform a classifier that has learned features based on more spurious correlations. We tested the trained classifiers on both photographs and paintings across the two classes using 1000 samples per domain.

In Table 4, the performance of the two classifiers are summarized. We found that both classifiers perform similarly well on the domain they are trained on. However, when the classifiers are tested on cross-domain data, we found that the painting-trained classifier performs better than the photo-trained classifier. This suggests that the classifier trained on paintings has learned a more generalizable feature representation for this task.

**Table 4:** Classifier performance across domains. Classifiers are trained to distinguish cotton/wool from silk/satin. The first column represents the classifier trained on photographs, and the second column represents the classifier trained on paintings. In the first row, the classifiers are tested on images of the same type they were trained on (i.e., trained and tested on photos, and trained and tested on paintings). In the second row, the classifiers are tested on the other medium, i.e., trained on photos and tested on paintings and vice versa.

	Photo → Photo	Painting → Painting
MEAN F1 Score	79.6%	80.5%
	Photo → Painting	Painting → Photo
MEAN F1 Score	49.5%	57.8%

We have reported the confusion matrices in Table 5. The photo classifier applied to paintings is heavily biased towards satin predictions. We hypothesize that this is because the photo classifier is relying on spurious cues (such as image background or clothing shape) over more robust cues and thus that the shift from photos to paintings causes its mispredictions. Only 21% of cotton samples are correctly identified as cotton while 79% are identified as satin. This skew in precision/recall across the classes is also reflected by the F1 scores for each class. On the other hand, the painting classifier applied to photos is much more balanced in its predictions, with 57-59% of predictions being correct. The precision/recalls are also much better balanced as reflected by the F1 scores.

**Table 5:** Confusion matrix for the two classifiers. The top represents the classifier trained on photos, tested on paintings which is heavily biased towards satin. The bottom represents the classifier trained on paintings, tested on photos, which is more balanced in its predictions.

Photo → Painting		
	Cotton	Satin
Cotton	20.83%	79.17%
Satin	9.72%	90.28%
Per class F1	31.91	67.01
Painting → Photo		
	Cotton	Satin
Cotton	58.82%	41.18%
Satin	42.86%	57.14%
Per class F1	55.56	60.00

*Human agreement with classifier cues*. How indicative are the cues used by each classifier to humans? We hypothesized that training networks on paintings might lead to the use of more perceptually relevant image features. If this is true, then the features used by the classifier trained on paintings should be preferably by humans.

**Fig 13:**
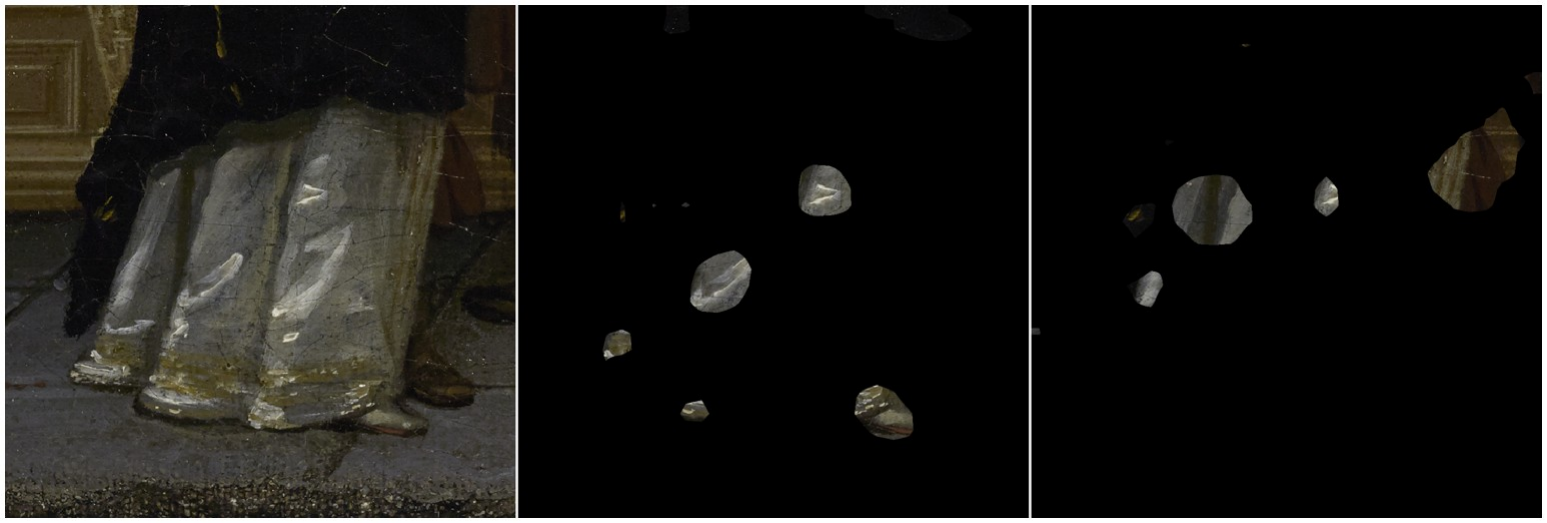
Visualization of cues used by classifiers. Left to right: Original Image, Masked Image (Painting Classifier), Masked Image (Photo Classifier). The unmasked regions represent evidence used by the classifiers for predicting “silk/satin” in this particular image. See main text for details. Image from *Interior of the Laurenskerk at Rotterdam*, by *Anthonie De Lorme, with figures attributed to Ludolf de Jongh*, 1662. Digital image courtesy of the Getty’s Open Content Program, reproduced under a CC0 license.

We produced evidence heatmaps with GradCAM [105] from the feature maps in the network before the fully connected classification layer. We extracted high resolution feature maps from images of size 1024 × 1024 (for a feature map of size 32 × 32). The heatmaps produced by GradCAM show which regions of an image the classifier uses as evidence for a specific class. If the cues (i.e., *evidence heatmaps*, such as in Fig 13) are clearly interpretable, this would imply the classifier has learned a good representation. For both models, we computed heatmaps for test images corresponding to their ground truth label. We conducted a user study on Amazon Mechanical Turk to find which heatmaps are judged as more informative by users. Users were shown images with regions corresponding to heatmap values that are above 1.5 standard deviations above the mean. Fig 13 illustrates an example. Users were instructed to “select the image that contains the regions that look the most like <material>”, where <material> was either cotton/wool or silk/satin. We collected responses from 85 participants, 57 of which were analyzed after quality control. For quality control, we only kept results from participants who spent over 1 second on average per trial.

**Fig 14:**
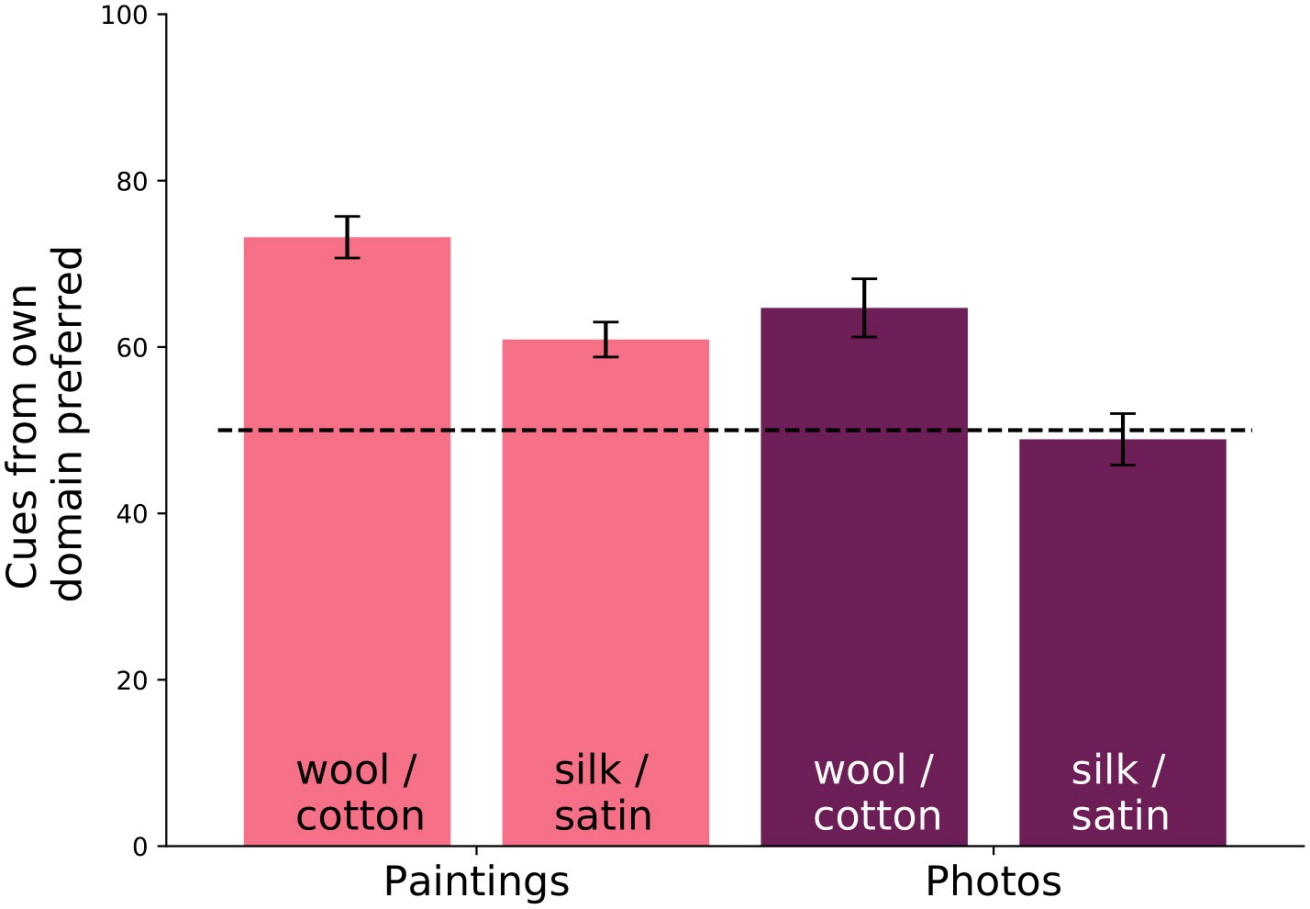
Human preference for classification cues used by each classifier. The y-axis represents how often humans prefer the cues from a classifier trained on the same domain as the test images. For example, the first bar indicates that in 73.2% of the cases, humans preferred cues from the classifier trained on paintings when classifying wool/cotton paintings (and thus, the inverse, that in 26.8% of the cases, humans preferred cues from the photo classifier.) Interestingly, note the last column—humans equally prefer cues used by both classifiers for classifying silk/satin photos despite the painting classifier never seeing a photo during training.

Overall, we found that the classifier trained on paintings uses evidence that is better aligned with evidence preferred by humans (Fig 14). This implies that training on paintings allows classifiers to learn more perceptually relevant cues, and it shows that this method might be useful to detect previously unknown perceptual cues.

Due to the domain shifts, training and testing a classifier on a single type of images will outperform a classifier trained and tested on different kind of images. Based on this, if paintings do not lead to a more robust feature representation we would expect the painting classifier to do best on paintings and the photo classifier to perform best on photos. Interestingly however, this does not hold when testing on photos of the satin/silk category (see last column of Fig 14). We found that users actually have no preference for the cues from either classifier, i.e., the cues from the painting classifier appears to be equally informative as the cues from the photo classifier for categorizing silk/satin in photos. This suggests that either (a) the painting classifier has learned human-interpretable perceptual cues for recognizing satin/silk, or (b) that the photo classifier has learned to classify satin/silk based on some spurious contextual signals that are difficult to interpret by humans. We asked users to elucidate their reasoning when choosing which set of cues they preferred. In general, users noted that they preferred the network which picks out regions containing the target class. Therefore, it seems that the network trained on paintings has learned better to distinguish fabric through the appearance of such fabrics in the image over other contextual signals (see Fig 13).

## Conclusion

In this paper, we presented the Materials in Paintings (MIP) dataset—a dataset of painterly depictions of different materials throughout time. The dataset can be visited, browsed and downloaded at materialsinpaintings.tudelft.nl. The MIP dataset consists of 19,325 high resolution images of painting. Unlike existing datasets that contains paintings, such as for example [75, 76], the MIP dataset contains exhaustive material labels across 15 categories for all paintings within the set. Additionally, human annotators have created 227,810 bounding boxes and we automatically identified an additional 94k bounding boxes. Each bounding box also contains a material label and half are additionally assigned with a fine-grained material label.

Although the findings reported in this study are valuable for their own sake, together they demonstrate the wide utility that a dataset of painterly depictions can serve. We hope that the MIP dataset can support research in multiple disciplines, as well as promote multidisciplinary research. We have shown that depictions in paintings are not just of interest for art history, but that they are also of fundamental interest for perception, as they can illustrate what cues the visual system may use to construct a perception. We have shown that computer vision algorithms trained on paintings appear to use cues more aligned with the human visual system, when compared to algorithms trained on photos. The benefits of this might also extend to learning perceptually robust models for image synthesis.

Our findings support our hope that the MIP dataset will be a valuable addition to the scientific community to drive interdisciplinary research in art history, human perception, and computer vision.
